# Transfer function of an asymmetric superconducting Gauss neuron

**DOI:** 10.3762/bjnano.16.85

**Published:** 2025-07-21

**Authors:** Fedor A Razorenov, Aleksander S Ionin, Nikita S Shuravin, Liubov N Karelina, Mikhail S Sidel’nikov, Sergey V Egorov, Vitaly V Bol’ginov

**Affiliations:** 1 Osipyan Institute of Solid State Physics RAS, Chernogolovka, Moscow District, 2 Academician Osipyan str., 142432, Russian Federation; 2 Moscow Institute of Physics and Technology, 9 Institutskiy per., Dolgoprudny, Moscow Region, 141701, Russian Federation; 3 Joint Venture Quantum Technologies, 121205, Moscow, Russian Federation

**Keywords:** Josephson interferometers, superconducting neural network, superconductivity

## Abstract

The Gauss neuron is a nonlinear signal converter, whose transfer function (TF) is described by the derivative of some sigmoidal dependence. A superconducting Gauss neuron can be implemented as a two-junction interferometer shunted symmetrically by an additional inductance. This work analyzes three cases of asymmetry that can occur in the experimental samples of Gauss neurons, that is, unequal critical currents of the interferometer’s Josephson junctions, asymmetric inductive shunting, and asymmetry of the input signal supply. We illustrate the modifications in equations and the shape of the TF compared to the symmetric case. The analysis performed provides an explanation for the key features observed in a previously conducted experiment.

## Introduction

Over the past decade, artificial neural networks have demonstrated their effectiveness and versatility in tasks related to processing large volumes of data, prediction, pattern recognition, and image and video generation. The increasing number of tasks and the growing volume of processed information highlight the relevance of using superconducting elements, which offer the advantages of high clock frequency and energy efficiency [1–2]. Studies [3–6] describe neuromorphic elements based on superconducting interferometers that emulate the signal response of biological neurons in various real-world scenarios. In [1,7–15], adiabatic neuromorphic interferometers were presented, whose energy consumption can be reduced to the fundamental limit of *kT*·ln 2 [16]. These devices contain one or two Josephson junctions (JJs) enclosed in a superconducting loop with three inductive elements. Such devices’ design is much simpler than that of a neuromorphic CMOS element, that contains about 20 transistors per cell [7–8], which also speaks in favor of superconducting neuromorphic devices.

**Figure 1:**
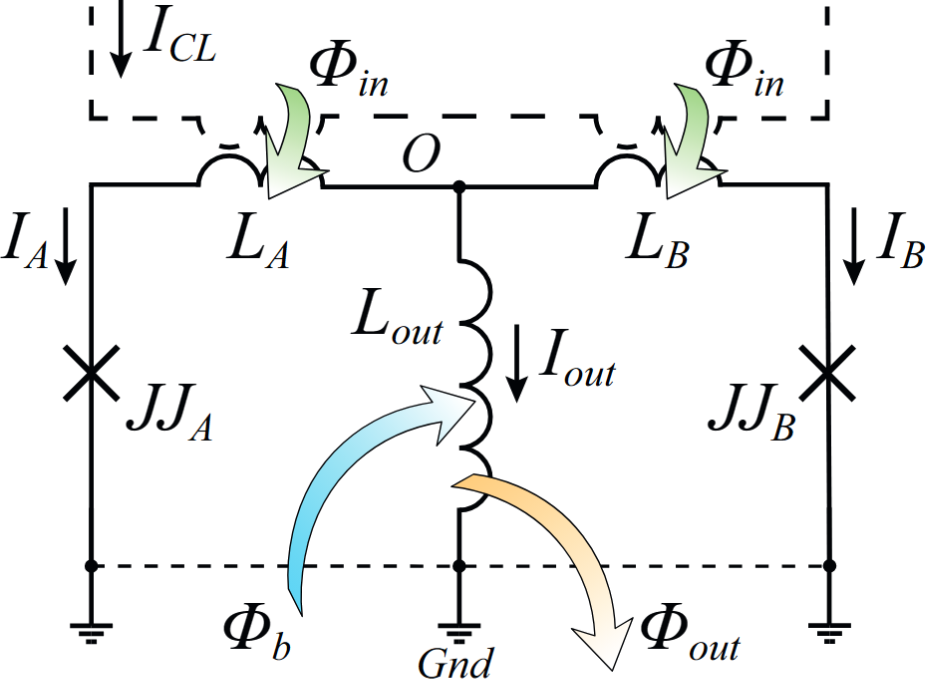
Schematic representation of a Gauss neuron, adapted from [14] (see details in the text).

The subject of this study is the Gauss neuron [1,7–9,11–12,14], schematically depicted in Figure 1. It consists of three arms connected at a common point O and grounded to a shared electrode (Gnd). Two arms (“Josephson” or “input” ones) each contain a Josephson junction JJ_A,B_ and an inductance *L*_A,B_, which is also used for receiving input signal. It is assumed that the input arms of the neuron are identical, including equal sensitivity to the input signal. These arms form the two-junction interferometer, and each of them is shunted by the third (output) arm. The latter consists of an inductive element *L*_out_, which generates a magnetic flux Φ_out_ = *L*_out_*I*_out_ when a current *I*_out_ flows through it (currents in the input arms are denoted as *I*_A,B_ in Figure 1). The input signal of the neuron is the magnetic flux Φ_in_, created using a control line (CL, shown as a dashed line in Figure 1), an external solenoid, or another method. An additional magnetic flux Φ_b_ is also introduced into the neuron, influencing the shape of the neuron’s transfer function (TF) [1,12,14].

When developing experimental superconducting neurons [17–18], it is essential to understand how the TF transforms when certain basic assumptions are violated. This can be important for improving device design and diagnosing potential faults. In this work, we consider three possible violations of the equivalence principle (“symmetry”) of the input arms of the Gauss neuron. The most expected violation is the imbalance of the critical currents of the JJs, i.e., *I*_cA_ ≠ *I*_cB_. Indeed, during the fabrication of Josephson devices, variations in critical currents of around 5% are observed, even among leading manufacturers [19]. We will refer to this violation as Josephson asymmetry (it can also be called critical current asymmetry or Josephson inductance asymmetry). Another possible violation involves asymmetry in the input arm inductances with *L*_A_ ≠ *L*_B_. This asymmetry may be referred to as “inductive” or “geometric” as it arises from differences in the shape of the input arms due to, for example, defects in the thin-film structure. The third type of asymmetry may be associated with unequal signal supply into the input arms of the neuron. Below, we analyze the transformation of the TF in each of these cases and compare it with experimental results [18].

## Symmetric Gauss Neuron

For clarity and systematic exposition, let us first consider the case of a symmetric Gauss neuron [8,12,14]. The equations of state consist of Kirchhoff’s law in the node O (Equation 1) and two phase balance equations in the partial loops of the neuron:


[1] $$I_{\text{cA}}\sin\varphi_{\text{A}}+I_{\text{cB}}\sin\varphi_{\text{B}}+I_{\text{out}}=0,$$

[2] $$\frac{\Phi_{0}}{2\pi }\varphi_{\text{A}}+L_{\text{A}}I_{\text{cA}}\sin\varphi_{\text{A}}+\Phi_{\text{in}}=\Phi_{\text{out}}+\Phi_{\text{b}},$$

[3] $$\frac{\Phi_{0}}{2\pi }\varphi_{\text{B}}+L_{\text{B}}I_{\text{cB}}\sin\varphi_{\text{B}}-\Phi_{\text{in}}=\Phi_{\text{out}}+\Phi_{\text{b}}.$$

Here, φ_A,B_ is the phase difference across the junctions JJ_A,B_, *I*_cA,B_ are the critical currents, and Φ_0_ is the magnetic flux quantum. Equation 2 is written for the left input loop, consisting of the left input and output arms (see Figure 1); Equation 3 is for the loop consisting of the right input and output arms (right input loop). The positive directions of currents (indicated by arrows in Figure 1) and the directions of loop traversal (counterclockwise and clockwise for the left and right loops, respectively) are chosen according to [14]. The phase balance equation for the full input loop, consisting of both input arms, is obtained by subtracting Equation 2–Equation 3. The symmetric case assumes *L*_A_ = *L*_B_ = *L* and *I*_cA_ = *I*_cB_ = *I*_c_.

The next step is to adopt dimensionless units: the magnetic flux is normalized by Φ_0_/2π, the current is normalized by the critical current *I*_c_, and the inductance is normalized by the Josephson inductance *L*_J_ = Φ_0_/2π*I*_c_. Thus,


[4] $$\frac{2\pi }{\Phi_{0}}\Phi_{\text{in}}=\phi_{\text{in}},\;\frac{2\pi }{\Phi_{0}}\Phi_{\text{out}}=\phi_{\text{out}},\;\frac{2\pi }{\Phi_{0}}\Phi_{\text{b}}=\phi_{\text{b}},$$

[5] $$L/L_{\text{J}}=l,\;L_{\text{out}}/L_{\text{J}}=l_{\text{out}},\;L_{\text{J}}=\Phi_{0}/2\pi I_{\text{c}}.$$

The normalized system of equations takes the form [14]:


[6] $$\sin\varphi_{\text{A}}+\sin\varphi_{\text{B}}+\phi_{\text{out}}/l_{\text{out}}=0,$$

[7] $$\varphi_{\text{A}}+l\sin\varphi_{\text{A}}+\phi_{\text{in}}=\phi_{\text{out}}+\phi_{\text{b}},$$

[8] $$\varphi_{\text{B}}+l\sin\varphi_{\text{B}}-\phi_{\text{in}}=\phi_{\text{out}}+\phi_{\text{b}}.$$

By solving this system for the input and output fluxes, we obtain the TF of the Gauss neuron ϕ_out_(ϕ_in_), which can be written as a two-parameter dependence:


[9] $$\phi_{\text{in}}=\frac{\varphi_{\text{B}}-\varphi_{\text{A}}}{2}+\frac{l}{2}\left(\sin\varphi_{\text{B}}-\sin\varphi_{\text{A}}\right),$$

[10] $$\phi_{\text{out}}=\frac{\varphi_{\text{A}}+\varphi_{\text{B}}}{2}+\frac{l}{2}\left(\sin\varphi_{\text{A}}+\sin\varphi_{\text{B}}\right)-\phi_{\text{b}},$$

[11] $$\phi_{\text{b}}=\frac{\varphi_{\text{A}}+\varphi_{\text{B}}}{2}+\left(l_{\text{out}}+\frac{l}{2}\right)\left(\sin\varphi_{\text{A}}+\sin\varphi_{\text{B}}\right).$$

Equation 10 and Equation 9 are derived as the sum and the difference of Equation 7 and Equation 8, respectively. Equation 11 is obtained by substituting Equation 10 into Equation 6 to eliminate the output signal ϕ_out_. By using the common method of introducing half-sum and half-difference of phases [1,8–9,12,14], φ_+_ = (φ_A_ + φ_B_)/2, φ_−_ = (φ_B_ − φ_A_)/2, the system of Equation 10 and Equation 11 can be represented as:


[12] $$\phi_{\text{in}}=\varphi_{-}+lg_{-},$$

[13] $$\phi_{\text{out}}=\frac{2l_{\text{out}}}{l+2l_{\text{out}}}\left(\varphi_{+}-\phi_{\text{b}}\right),$$

[14] $$\frac{\phi_{\text{b}}-\varphi_{+}}{l+2l_{\text{out}}}-g_{+}=0,$$

where, for brevity, we have introduced the notation *g*_±_ = sinφ_±_cosφ_∓_. Equation 12 and Equation 13 determine the dependence of the input and output fluxes on the parameters φ_±_, while Equation 14 links these parameters. One may also note the linear relationship between the output signal and the mean phase φ_+_ according to Equation 13.

**Figure 2:**
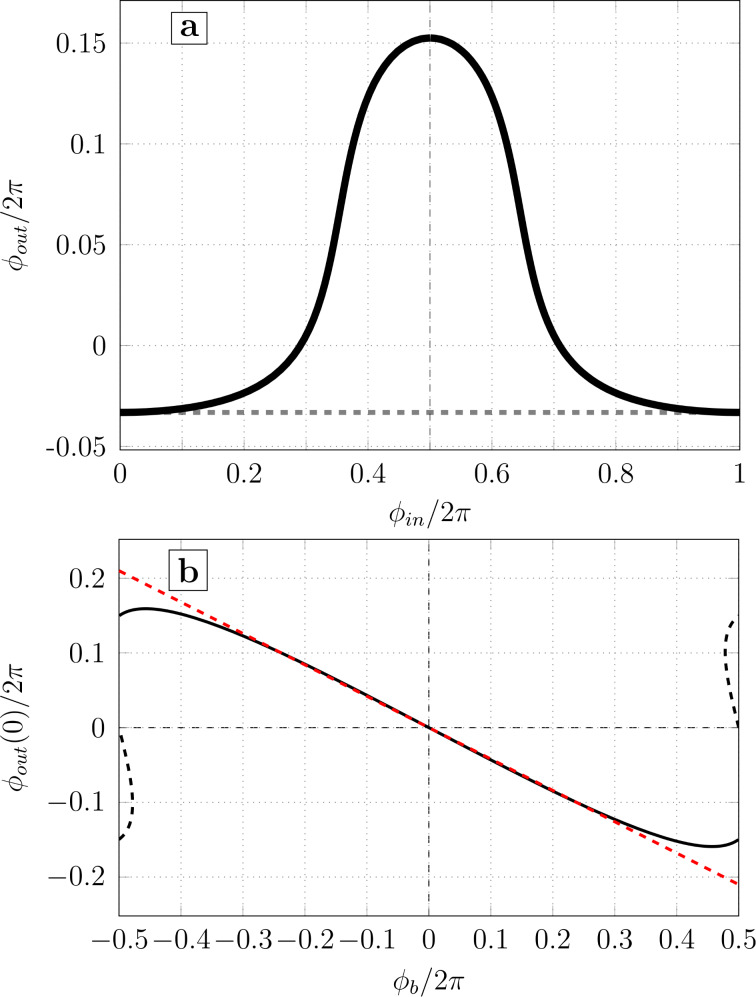
(a) Transfer function of the symmetric Gauss neuron according to Equation 12–Equation 14 for *l* = 0.29, *l*_out_ = 0.48, and ϕ_b_ = 0.155π. The calculation parameters correspond to the experimental work [18]. (b) Dependence of the baseline ϕ_out_(0) on the bias flux ϕ_b_ for the same sample (solid black line). The red dashed line shows the linear approximation of the central part of the dependence (see discussions in Sections *Symmetric Gauss Neuron* and *Results (C)*). The black dashed line shows the secondary solution that does not provide a bell-shaped response.

The TF (Equation 12–Equation 14) is obviously periodic with respect to ϕ_in_. Within the first period, the TF of the Gauss neuron represents a symmetric bell-shaped curve that “rests” on a horizontal line (Figure 2a). The symmetry of the TF allows for the use of such neurons in radial basis function networks [20], with the position of the baseline being taken as the zero value of the TF when designing neural networks based on this element. This value can be determined from the system of Equation 12–Equation 14 as the value of ϕ_out_ at zero ϕ_in_. By symmetry, we obtain φ_−_ = 0 according to Equation 12 and


[15] $$f\left(\phi_{\text{out}}\right)=\frac{\phi_{\text{out}}}{2l_{\text{out}}}+\sin\left(\phi_{\text{b}}+\frac{l+2l_{\text{out}}}{2l_{\text{out}}}\phi_{\text{out}}\right)=0,$$

according to Equation 13 and Equation 14. The solution of this transcendental equation can be represented as a parameterized integral [8]:


[16] $$\phi_{\text{out}}\left(0\right)=\int_{0}^{-\pi \text{sgn}\phi_{\text{b}}}\theta \left[f\left(\phi_{\text{out}}\right)\text{sgn}\left(\phi_{\text{b}}\right)\right]\text{d}\phi_{\text{out}},$$

where θ(*x*) is the Heaviside step function. The numerical solution of Equation 15 is shown in Figure 2b. The solution is 2π-periodic, and the dashed black line depicts the secondary branch of the solution that does not allow for a bell-shaped TF and cannot be obtained from Equation 16. Such a solution appears for sufficiently large ϕ_b_ when (*l* + 2*l*_out_) *>* 1. The calculation parameters correspond to the experimental sample investigated in our previous work [18]. It can be seen that in a sufficiently wide range, the graph is close to linear: deviations from the linear approximation are observed only for sufficiently large 
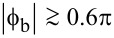
.

## Results

### A. Josephson asymmetry

Now, let us assume that, for whatever reason, the critical currents of the neuron’s JJs have become unequal, *I*_cA_ ≠ *I*_cB_. First of all, a difficulty arises when introducing dimensionless parameters in the system of Equation 1–Equation 3 as it is unclear which value of *I*_cA,B_ should be used for normalization in Equation 5. We begin by normalizing the magnetic flux and dividing Equation 2 and Equation 3 (which have the corresponding dimensions) by the quantity Φ_0_/2π. They will immediately take the normalized form (similar to Equation 7 and Equation 8) if we define


[17] $$l_{\text{A},\text{B}}=\frac{L}{L_{{\text{J}}_{\text{A},\text{B}}}},\;L_{{\text{J}}_{\text{A},\text{B}}}=\frac{\Phi_{0}}{2\pi I_{\text{cA},\text{B}}}.$$

Thus, it can be said that in the case of Josephson asymmetry, the system of Equation 1–Equation 3 allows for the normalization of the inductances of the input arms to individual Josephson inductances 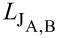. Note that the quantities *l*_A,B_ can also be introduced in Equation 1. To do this, we multiply it by the inductance *L* and notice that *LI*_cA,B_sinφ_A,B_ are the magnetic fluxes created by the Josephson currents in the elements *L*_A,B_. Therefore, the resulting equation should also be divided by the unit of magnetic flux Φ_0_/2π. The normalized system of equations thus takes the form


[18]

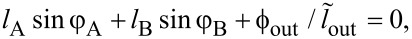




[19] $$\varphi_{\text{A}}+l_{\text{A}}\sin\varphi_{\text{A}}+\phi_{\text{in}}=\phi_{\text{out}}+\phi_{\text{b}},$$

[20] $$\varphi_{\text{B}}+l_{\text{B}}\sin\varphi_{\text{B}}-\phi_{\text{in}}=\phi_{\text{out}}+\phi_{\text{b}},$$

where 
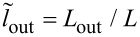
. By performing transformations similar to those in Section *Symmetric Gauss Neuron*, we obtain


[21] $$\phi_{\text{in}}=\frac{\varphi_{\text{B}}-\varphi_{\text{A}}}{2}+\frac{1}{2}\left(l_{\text{B}}\sin\varphi_{\text{B}}-l_{\text{A}}\sin\varphi_{\text{A}}\right),$$

[22] $$\phi_{\text{out}}=\frac{\varphi_{\text{B}}+\varphi_{\text{A}}}{2}+\frac{1}{2}\left(l_{\text{B}}\sin\varphi_{\text{B}}+l_{\text{A}}\sin\varphi_{\text{A}}\right)-\phi_{\text{b}},$$

[23]

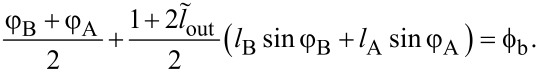



The transition to the half-sum and half-difference of phases in Equation 21–Equation 23 naturally exposes the “asymmetry angle” α according to


[24] $$\tan\alpha =\frac{l_{\text{A}}}{l_{\text{B}}}=\frac{I_{\text{cA}}}{I_{\text{cB}}}.$$

The normalized inductances of the arms are expressed through the asymmetry angle as follows:


[25]

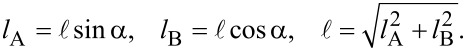



Thus, the parameter 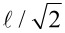 characterizes the effective inductance of the input circuit, and tanα represents the imbalance of the critical currents. Introducing (φ_+_, φ_−_) and performing some simple trigonometric transformations, we obtain the two-parameter solution in the form:


[26]

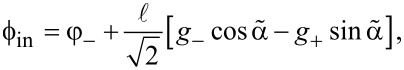




[27]

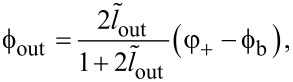




[28]

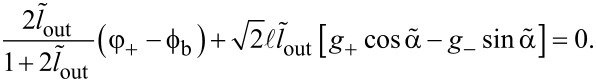



Here, for brevity, we introduce the notation 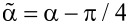. In general, the system of Equation 26–Equation 28 resembles the form of the solution in Equation 12–Equation 14 with the exception of the terms containing 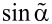. Equation 27 coincides with Equation 13. This is possible because in Equation 22 and Equation 23, the coefficients before the sine terms in the parentheses are the same. Josephson asymmetry leads to the replacement of *g*_+_ and *g*_−_ in Equation 12–Equation 14 with linear combinations of the *g*_±_ terms, as indicated in the square brackets in Equation 26–Equation 28. The functions of Equation 12–Equation 14 and Equation 26–Equation 28 coincide when α = π/4, which occurs in the symmetric case *I*_cA_ = *I*_cB_. The range of variation for the parameter 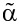 is ±π/4 when, for example, *I*_cA_ changes within 0 ≤ *I*_cA_ *<* ∞. Exceeding these limits is possible if one of the Josephson junctions is a π-junction with a negative sign of the current–phase relation (see, for example, [21–22]). The use of π-junctions in the context of developing adiabatic Josephson logic is discussed, for example, in [23–24].

**Figure 3:**
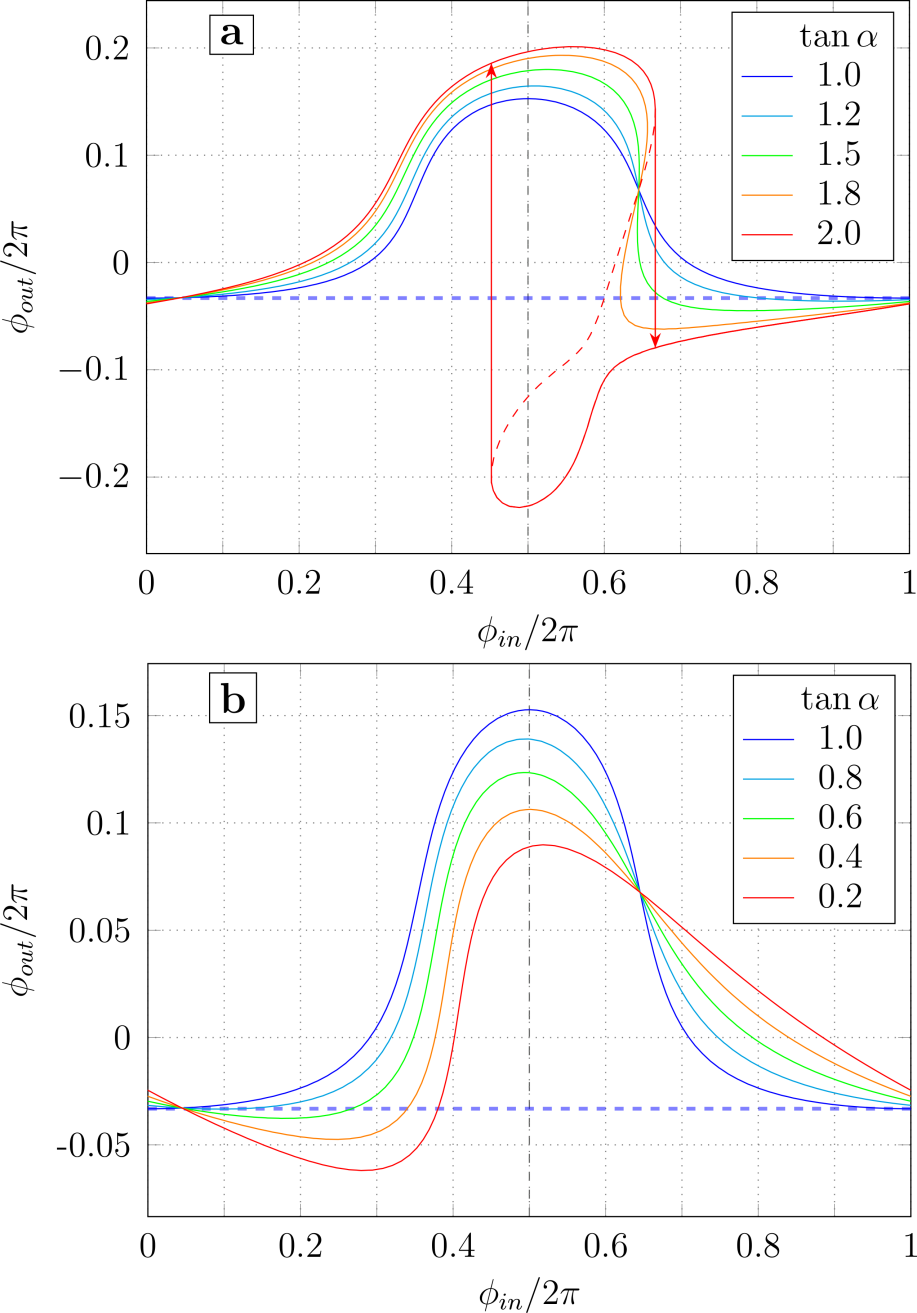
Transfer function of the Gauss neuron according to Equation 26–Equation 28 at different values of the Josephson asymmetry parameter tanα = *I*_cA_/*I*_cB_ for tanα ≥ 1 (panel a) and tanα ≤ 1 (panel b). The parameters are 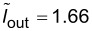, *l*_B_ = 0.29, and ϕ_b_ = 0.155π.

Figure 3 shows the calculated TF for different cases of Josephson asymmetry. The calculation parameters (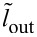, *l*_B_, ϕ_b_) were chosen according to the experimental work in [18]. It is assumed that the parameter *I*_cA_ changes while *I*_cB_ remains constant. It can be seen that as tanα increases, the curve becomes asymmetric, that is, the left part becomes flatter, and the right one steeper (Figure 3a). For sufficiently large asymmetry (
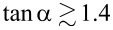
 for the parameters in Figure 3a), the transfer function becomes multivalued, and hysteresis should be observed during the experiment. In the case of reverse asymmetry (tanα *<* 1), the right wing of the transfer function becomes flatter (Figure 3b). The baseline remains horizontal (ϕ_out_(0) = ϕ_out_(2π)), but it may intersect the distorted transfer function. Note that a significant distortion of the bell-shaped transfer function is observed when the critical currents diverge by a factor of 1.3 or more, while an asymmetry of the order of 1.05 is unlikely to be noticeable to the naked eye. The difference between Figure 3a and Figure 3b is due to the different meaning of zero and infinite tanα limits: the first one corresponds to the break of the junction JJ_A_, while the second corresponds to shorting of the junction JJ_A_. In the first case, the inductance *l*_A_ turns to infinity and the neuron becomes a single-junction SQUID, whose multistability condition is 
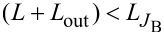
. The screening current circulates mainly in the JJ_B_–*L*–*L*_out_ partial loop. In the case of infinite tanα, the Gauss neuron becomes a shunted single-junction interferometer (in fact, a Sigma neuron [7]), whose multistabitity condition can be expressed as (*L* + LL_out_/(*L* + *L*_out_)) *< L*_J_ (see [13,17]). In that case, the screening current circulates mainly in the JJ_A_–*L*–*L*_out_ circuit, which defines the side the TF is tilted to.

Characterizing the Josephson asymmetry through the ratio of critical currents or the angle α is not the only possible approach. Using the definitions in Equation 24 and Equation 25, we get:


[29]

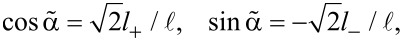



where *l*_±_ are defined as:


[30]

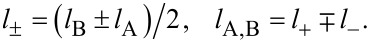



Using these definitions (and also the definition of the coefficient 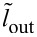), the transfer function of the Gauss neuron with Josephson asymmetry takes the form:


[31] $$\phi_{\text{in}}=\left[\varphi_{-}+l_{+}g_{-}\right]+l_{-}g_{+},$$

[32]

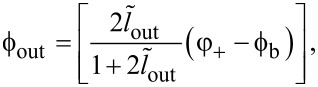




[33]

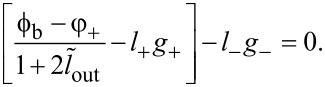



The form of Equation 31–Equation 33 is closest to Equation 12–Equation 14 (the matching terms are highlighted in square brackets). The parameter *l*_+_ characterizes the effective inductance of the input circuit, while *l*_−_ represents the imbalance in the normalized inductances of the Josephson circuits. Note the complete coincidence of Equation 13 and Equation 32, which define the linear relationship between the output signal and the sum phase. The influence of Josephson asymmetry reduces to the appearance of conjugate terms of the form *l*_−_*g*_±_ in Equation 12 and Equation 14. The transition to the symmetric case occurs when *l*_A_ = *l*_B_ = *l*_+_, *l*_−_ = 0.

### B. Inductive asymmetry

Now let us consider the case of asymmetry in the self-inductances *L*_A_ ≠ *L*_B_ (“inductive asymmetry”). We will assume that the Josephson inductances are the same: 
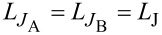
. This allows us to apply the standard normalization of the inductances of the Gauss neuron elements described in Section *Symmetric Gauss Neuron*. The normalized equations of the states described in Equation 1–Equation 3 take the form


[34] $$\sin\varphi_{\text{A}}+\sin\varphi_{\text{B}}+\phi_{\text{out}}/l_{\text{out}}=0,$$

[35] $$\varphi_{\text{A}}+l_{\text{A}}\sin\varphi_{\text{A}}+\phi_{\text{in}}=\phi_{\text{out}}+\phi_{\text{b}},$$

[36] $$\varphi_{\text{B}}+l_{\text{B}}\sin\varphi_{\text{B}}-\phi_{\text{in}}=\phi_{\text{out}}+\phi_{\text{b}}.$$

It differs from the “symmetric” system (Equation 6–Equation 8) only by the different values of inductances *l*_A_ and *l*_B_ in Equation 35 and Equation 36. By adding and subtracting Equation 35 and Equation 36, we obtain the system of equations in the following form:


[37] $$\phi_{\text{in}}=\frac{\varphi_{\text{B}}-\varphi_{\text{A}}}{2}+\frac{1}{2}\left(l_{\text{B}}\sin\varphi_{\text{B}}-l_{\text{A}}\sin\varphi_{\text{A}}\right),$$

[38] $$\phi_{\text{out}}=\frac{\varphi_{\text{B}}+\varphi_{\text{A}}}{2}-\phi_{\text{b}}+\frac{1}{2}\left(l_{\text{B}}\sin\varphi_{\text{B}}+l_{\text{A}}\sin\varphi_{\text{A}}\right),$$

[39] $$\frac{\varphi_{\text{B}}+\varphi_{\text{A}}}{2}+\left(\left(l_{\text{out}}+\frac{l_{\text{B}}}{2}\right)\sin\varphi_{\text{B}}+\left(l_{\text{out}}+\frac{l_{\text{A}}}{2}\right)\sin\varphi_{\text{A}}\right)=\phi_{\text{b}}.$$

A transition to phases φ_+_ and φ_−_ is hindered by the fact that the coefficients in front of the Josephson currents sinφ_A,B_ in Equation 39 differ from the coefficients in Equation 37 and Equation 38 (unlike in the system of Equation 21–Equation 23). In this case, using the asymmetry angle appears unreasonable. By introducing the quantities *l*_±_ according to the definition in Equation 30, we obtain the following system after simple transformations:


[40] $$\phi_{\text{in}}=\left[\varphi_{-}+l_{+}g_{-}\right]+l_{-}g_{+},$$

[41] $$\phi_{\text{out}}=\frac{2l_{\text{out}}}{l_{+}+2l_{\text{out}}}\left(\left[\varphi_{+}-\phi_{\text{b}}\right]+l_{-}g_{-}\right),$$

[42] $$\left[\frac{\phi_{\text{b}}-\varphi_{+}}{l_{+}+2l_{\text{out}}}-g_{+}\right]-\frac{l_{-}}{l_{+}+2l_{\text{out}}}g_{-}=0.$$

The terms inside square brackets are those present in the “symmetric” system (Equation 12–Equation 14). Note that in the case of inductive asymmetry, the linear relationship between ϕ_out_ and φ_+_ is not preserved (unlike in the Josephson asymmetry case). The condition relating the parameters of the phase differences φ_±_ is also different (compared to the system of Equation 31–Equation 33). The symmetric case is obtained when *l*_A_ = *l*_B_ = *l*_+_, and *l*_−_ = 0.

**Figure 4:**
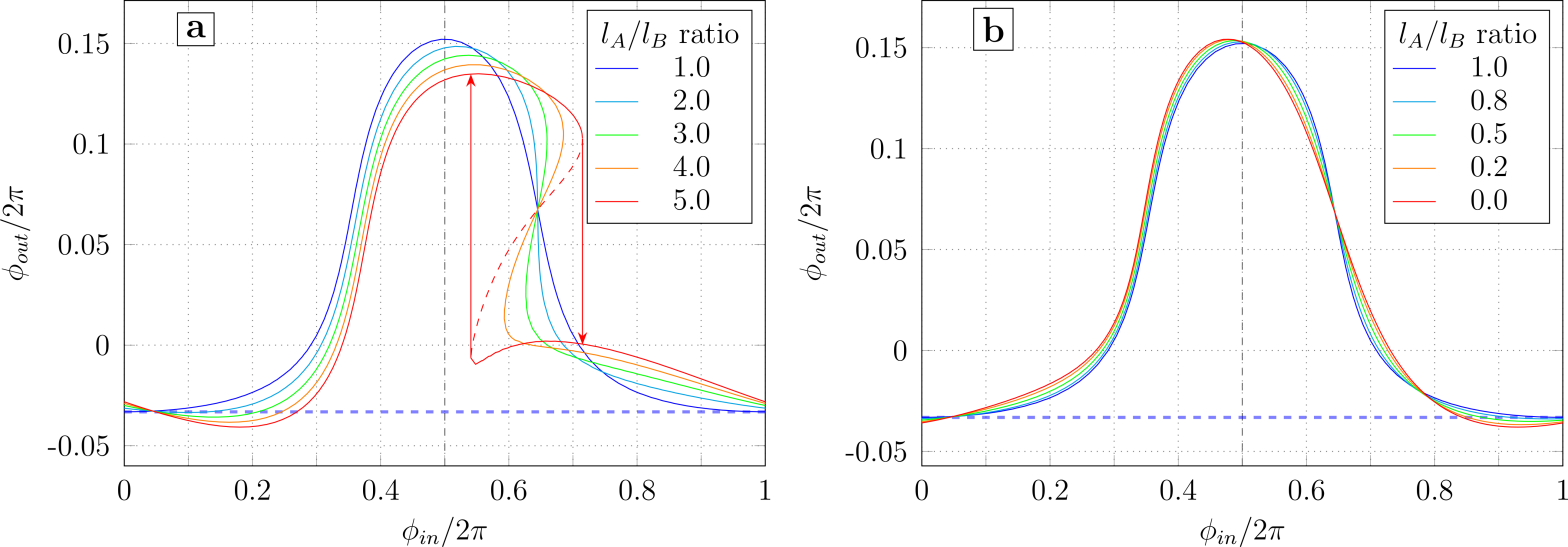
Transfer function of the Gauss neuron with different inductance ratios for the receiving arms (given in the legend) at a constant value of *l*_B_ in case of increasing (panel a) or decreasing (panel b) *l*_A_.

Figure 4 shows the family of transfer functions for different values of *l*_A_/*l*_B_. The calculation parameters (*l*_B_, *l*_out_, ϕ_b_) correspond to the sample studied experimentally in [18]. It is assumed that *L*_A_ changes while *L*_B_ remains constant. As in the previous section, inductive asymmetry causes a tilt of the transfer function, bending one wing of the transfer function and widening the other. As *l*_A_/*l*_B_ increases, the distortion of the transfer function increases (Figure 4a), which leads to its multivaluedness (and hence to hysteresis). This is related to the increase in the inductance of the overall receiving circuit 2*l*_+_ as *l*_A_ increases. The opposite change (reducing *l*_A_ while keeping *l*_B_ constant) weakly affects the shape of the transfer function, mainly leading to a slight distortion of the right half of the graph (Figure 4b). The difference between Figure 4a and Figure 4b can be understood by analogy with Josephson asymmetry. Note that all the distortions in Figure 3 and Figure 4 have slightly different shapes, which allows them to be distinguished during the initial analysis of experimental data.

In recent years, a number of superconducting devices have been proposed based on very thin superconducting films whose kinetic inductance may be comparable to the magnetic one [11,25–26]. Indeed, the inductance of a superconducting film carrying an electric current consists of two components, namely, the magnetic inductance (originating from the magnetic field energy) and the kinetic inductance (originating from the kinetic energy of the superconducting electrons). Should one want to account for the kinetic inductance, the initial Equation 1–Equation 3 remain unchanged, as it is the total inductance value that determines the phase balance conditions in Equation 2 and Equation 3. However, the value of ϕ_out_ in Equation 13 has then the meaning of the phase difference across the output arm, which cannot be directly measured in an experiment. The measurable output signal is defined only by the component of ϕ_out_ that originates from the magnetic flux generated by the output current *I*_out_. To account for this, one can simply rescale ϕ_out_ in Equation 2 and Equation 3 by the factor 
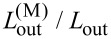
, where 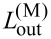 is the magnetic part of total inductance *L*_out_. Therefore, the use of ultrathin superconducting films is not a promising approach for implementing a superconducting Gauss neuron.

### C. Input asymmetry

One more possible type of asymmetry is related to the unequal input signal supply to the neuron’s receiving arms. To parameterize this asymmetry, we introduce the parameter *t*, such that the magnetic fluxes in the left and right partial loops of the neuron are (1 ± *t*)Φ_in_. Then the total flux in the neuron is 2Φ_in_ (as in previous sections), and 
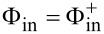
 is simply the half-sum of the input fluxes in the partial loops. The imbalance (half-difference) of the input fluxes is the asymmetry term 
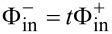
. In a practical situation, the magnetic flux is supplied into the neuron via a CL, inductively coupled to the receiving elements in some manner. Therefore, input asymmetry effectively means that the mutual inductances 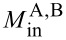 between the CL and the Josephson arms of the neuron are different. In this case, we can express the coefficient *t* through these inductances. Writing the input fluxes in the partial loops as 
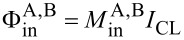
 (where *I*_CL_ is the current in the CL), we get


[43] $$t=\frac{\Phi_{\text{in}}^{-}}{\Phi_{\text{in}}^{+}}=\frac{M_{\text{in}}^{\text{A}}-M_{\text{in}}^{\text{B}}}{M_{\text{in}}^{\text{A}}+M_{\text{in}}^{\text{B}}}.$$

Let us assume that the arms of the neuron are symmetric, meaning that there is no inductive or Josephson asymmetry. Then, the system of equations of state for the Gauss neuron in the standard normalization can be written as


[44] $$l_{\text{out}}\left(\sin\varphi_{\text{A}}+\sin\varphi_{\text{B}}\right)+\phi_{\text{out}}=0,$$

[45] $$\varphi_{\text{A}}+l\sin\varphi_{\text{A}}+\left(1+t\right)\phi_{\text{in}}=\phi_{\text{out}}+\phi_{\text{b}},$$

[46] $$\varphi_{\text{B}}+l\sin\varphi_{\text{B}}-\left(1-t\right)\phi_{\text{in}}=\phi_{\text{out}}+\phi_{\text{b}}.$$

Upon transformations analogous to those made in Section *Symmetric Gauss Neuron*, the first equation of the new system (compare with the system of Equation 12–Equation 14) remains unchanged. The other two acquire new terms proportional to the asymmetry term *t*ϕ_in_:


[47] $$\phi_{\text{in}}=\left[\varphi_{-}+lg_{-}\right],$$

[48] $$\phi_{\text{out}}=\left[\frac{2l_{\text{out}}}{l+2l_{\text{out}}}\left(\varphi_{+}-\phi_{\text{b}}\right)\right]+\frac{2l_{\text{out}}}{l+2l_{\text{out}}}t\phi_{\text{in}},$$

[49] $$\left[\frac{\phi_{\text{b}}-\varphi_{+}}{l+2l_{\text{out}}}-g_{+}\right]-\frac{1}{l+2l_{\text{out}}}t\phi_{\text{in}}=0.$$

One may note the mixing of the asymmetry term to the output flux according to Equation 47–Equation 49. If *t* = 0, the system of Equation 47–Equation 49 transforms into Equation 12–Equation 14.

**Figure 5:**
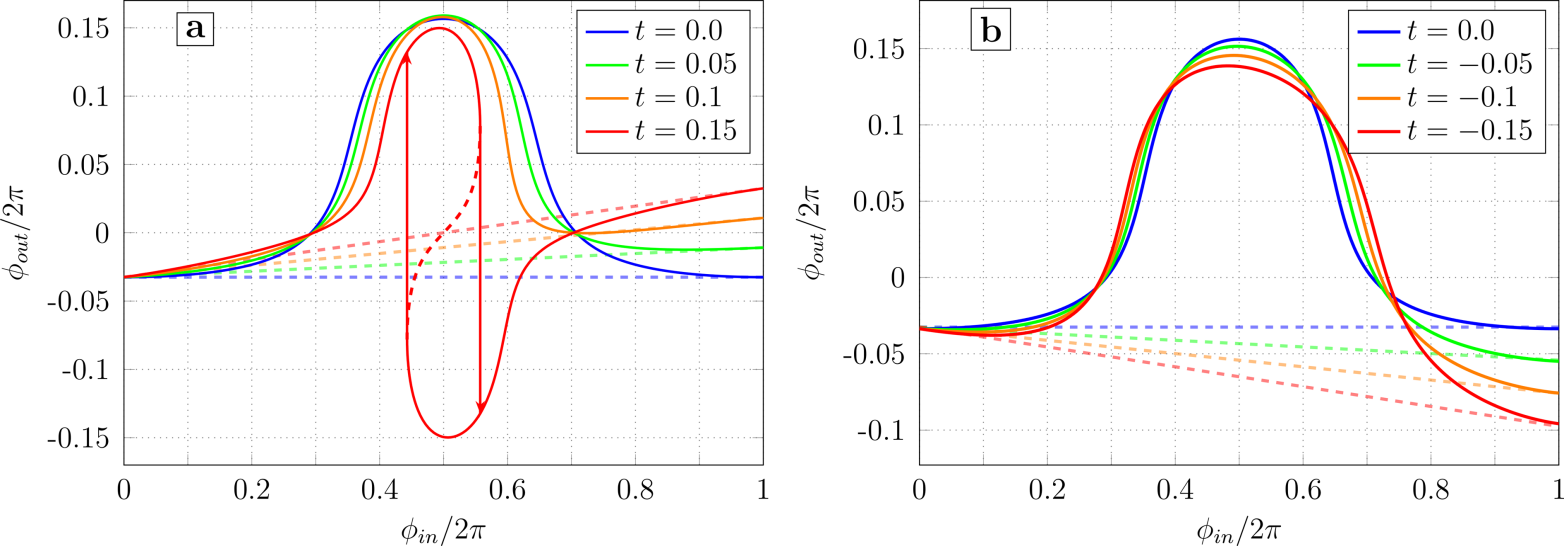
Transfer function of the neuron according to Equation 47–Equation 49 with different imbalance coefficients of the input signal *t* (positive on panel a and negative on panel b). Dashed lines represent the asymptotic baselines.

The TF plots for different values of the asymmetry parameter *t* are shown in Figure 5. The calculation parameters (*l*, *l*_out_, ϕ_b_) correspond to the sample studied experimentally in [18]. For *t* = 0, the TF is a (blue) bell on a horizontal baseline, as demonstrated in Section *Symmetric Gauss Neuron*. However, for non-zero *t*, the baseline becomes slanted and the transfer function essentially acquires a “linear component”. This behavior can be understood by noticing that the input signal is essentially “mixed” into the bias flux: to obtain Equation 47–Equation 49 from Equation 12–Equation 14, one should make a substitution ϕ_b_→ϕ_b_ − *t*ϕ_in_. This can be seen in Equation 44–Equation 46 by moving the asymmetry terms to the right-hand side. In other words, when ϕ_in_ is swept in the positive direction, the effective bias flux 
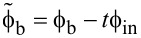
 (which determines the imbalance of the magnetic flux in the receiving loops ofthe Gauss neuron) decreases for *t >* 0. The decrease in 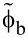, in turn, leads to a shift in the TF’s baseline value linearly with 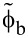 (Figure 2b) for sufficiently small 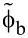. Linearity requires correspondingly small *t* (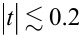 for Figure 5) since within one period of the input signal, the shift of ϕ_b_ reaches 2π*t*.

The increase in the slope of the baseline as *t* grows (in absolute value) makes the left branch of the transfer function (ϕ_in_ ≤ 0.5) flatter, and the right branch (ϕ_in_ ≥ 0.5) steeper. As *t* increases, the right branch becomes vertical, and at *t* ≈ 0.13, the transfer function becomes hysteretic (see the red curve in Figure 5a). The slope of the linear component is inverted when the sign of *t* is changed (Figure 5b).

## Discussion

**Figure 6:**
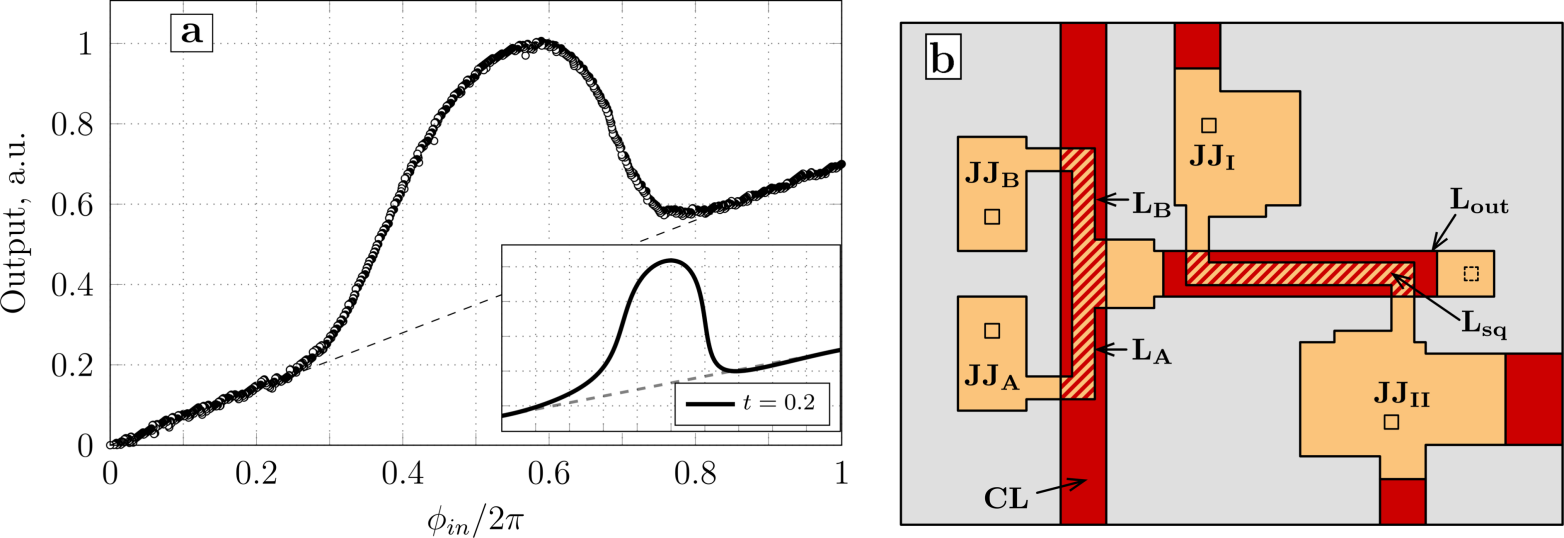
(a) Experimental transfer function of the superconducting Gauss neuron prototype according to [18]. The dashed lines show the baseline (see discussion in Sections *Symmetric Gauss Neuron* and *Results (C)*). The inset shows the calculated TF with *t* = 0.2 and ϕ_b_ = 0.3π. The dashed lines show the baseline (see discussion in Sections *Symmetric Gauss Neuron* and *Results (C)*). (b) Schematic of the structure of the studied sample. *L*_A,B_ denote receiving arms, *L*_out_ is the output arm, JJ_A,B_ are the JJs of the neuron, JJ_I,II_ are the JJs of the measuring element (i.e.,SQUID), and *L*_sq_ is the loop of the measuring element. Different colors represent elements in different layers of the multilayer structure. Hatching indicates the areas of inductive coupling with the input and readout elements. The boundaries of the drawing coincide with the boundaries of the superconducting screen, shown in gray.

All the asymmetry types considered are “independent”, meaning they cannot be reduced to one another through algebraic transformations. This statement is evident for input asymmetry, which leads to a slope of the baseline, unlike the other two cases. As for Josephson and inductive asymmetries, the corresponding equations of state (Equation 18–Equation 20 and Equation 34–Equation 36) differ only in the form of the first equation in the system (which originates from Kirchhoff’s law) and can be reduced to a common form only in the case *l*_A_ = *l*_B_ (that is, for a symmetric Gauss neuron). Moreover, the three types of symmetry breaking for the Gauss neuron presented here exhaust the list of possible asymmetries of its arms. Indeed, each receiving arm of the Gauss neuron (Figure 6) is formed by two elements (a JJ and an inductance) and is characterized by three quantities, namely, its own (geometric) inductance, the critical current of the JJ (Josephson inductance), and the sensitivity to the input signal (i.e., mutual inductance with the CL). The fluxes ϕ_out_ and ϕ_b_ cannot be a direct source of asymmetry in our model, since they are generated through a single element *L*_out_, common to both receiving loops. Nevertheless, *L*_out_ can lead to an effective asymmetry of the input signal supply, as will be shown below.

Let us try to apply the results obtained above to the experimental data presented in our work [18]. The experimental curve (see Figure 6a) represents a flat bell over a slanted baseline, which indicates the presence of input asymmetry. This is surprising because both receiving areas of the Gauss neuron are identical in shape (Figure 6b). However, the effective input asymmetry may arise due to direct interaction of the input and readout elements (which does not involve the neuron as a non-linear converter) as was shown in [27]. Despite the use of a superconducting screen in experiments [17–18], such an interaction can occur due to the finite size of the screen. The interaction is mediated by circulating currents in the screen, which may be non-zero even at a significant distance from the CL [17,28].

To take this effect into consideration, one should consider the method of measuring the output flux Φ_out_ by stabilizing the magnetic flux Φ_sq_ via the measuring SQUID. The latter consists of an inductive element *L*_sq_, closed onto a superconducting screen through JJs JJ_I,II_ (an asymmetric two-junction SQUID, Figure 6b). The output signal is the current in the feedback loop of the SQUID *I*_fb_ that compensates the change in the output flux while sweeping Φ_in_. In other words, the current 
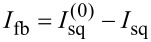
 represents the difference between the initial and current values of the current flowing through the loop of the measuring element. So, the transfer function of the experimental sample has a somewhat different (“current”) representation compared to the earlier proposed (“flux”) one. The relationship between *I*_fb_ and Φ_out_ is given by


[50] $$\Phi_{\text{out}}=L_{\text{out}}I_{\text{out}}+M_{\text{out}}\left(I_{\text{sq}}^{\left(0\right)}-I_{\text{fb}}\right),$$

where *M*_out_ is the mutual inductance between the SQUID and the output element. The variable *I*_out_ can be eliminated from Equation 50 using the invariance condition for the magnetic flux in the SQUID [17]:


[51] $$\Phi_{\text{sq}}=M_{\text{out}}I_{\text{out}}+L_{\text{sq}}I_{\text{sq}}+M_{\text{sq}}I_{\text{CL}}=\text{const}\left(I_{\text{CL}}\right).$$

After some straightforward transformations, one can obtain:


[52] $$\Phi_{\text{out}}=\frac{L_{\text{sq}}L_{\text{out}}^{*}}{M_{\text{out}}}I_{\text{fb}}+\left[\frac{L_{\text{out}}}{M_{\text{out}}}\Phi_{\text{sq}}-\frac{L_{\text{sq}}L_{\text{out}}^{*}}{M_{\text{out}}}I_{\text{sq}}^{\left(0\right)}\right]-\frac{L_{\text{out}}M_{\text{sq}}}{M_{\text{out}}}I_{\text{CL}},$$

where


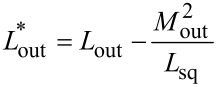


is the inductance of the output element renormalized due to the interaction with the reading element [17–18,27].

Equation 52 defines the relation between “flux-type” and “current-type” output signals. It is linear but contains three terms. The first one illustrates a linear type of *I*_fb_(Φ_out_) dependence. The second one represents a “shift term” that ensures a non-zero value of the bias flux even if Φ_b_ = 0. This can be verified by substituting Equation 52 into Equation 2 and Equation 3. Note that no special signal line to provide a bias flux into output inductance was realized in the experimental work [18], which, however, did not prevent us from observing a noticeable output signal. However, the effective bias is hard to control during the experiment, so it was estimated as a fitting parameter in [18]. Finally, the third term in Equation 52 arises due to the direct interaction between the input (CL) and readout (SQUID) elements (Equation 51). Upon substitution into Equation 2–Equation 3, the third term will cause the appearance of terms characteristic of input asymmetry with *t* = *L*_out_*M*_sq_/*M*_out_*M*_in_ (with 
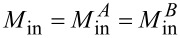
). Substituting further the values *L*_out_ = 7.2 pH, *M*_sq_ = 0.1 pH, *M*_out_ = 2.7 pH, and *M*_in_ = 2.4 pH given in [17–18], we get *t* = 0.2. Calculations based on Equation 52 give a similar shape of the TF (compared to Figure 6a) at ϕ_b_ = 0.3π. Thus, experimental results correspond to the expected ones, and a quantitative analysis shall be the subject of our subsequent publications.

The problem with cross-talk mentioned above (i.e., screen-mediated interaction) may become more and more severe when one connects more neurons together. This is why the search for the ways to suppress the cross-talk remains one of the main directions of the neuron’s design optimization. It is worth noting that the expression for the *t*-factor implies that it is possible to change its value by changing *L*_out_. However, the change of the output arm’s length is not the best way to suppress the input asymmetry. Indeed, the unlimited decrease in *L*_out_ is impossible at constant values of *M*_out_ as its length cannot be smaller than the overlap region with the SQUID-sensor loop. Therefore, the *t*-factor can be just increased with no practical meaning. The most promising ways to dump the effective input asymmetry are the increase of input mutual inductance *M*_in_ and the suppression of the screen-mediated interaction (a decrease of the *M*_sq_ value). Some methods of suppressing this interaction are discussed in [26]. The simplest ones include increasing the size of the screen and creating a reverse CL that is not coupled to the neuron (except screen-mediated coupling) and carries the control current in the opposite direction. Calculations show that this decreases *M*_sq_ by about five times. It is also useful to eliminate sections of the SQUID that are parallel to the CL. This is the main direction of optimization of the Gauss neuron design at the present time.

## Conclusion

In this work, the changes in the form of the transfer function (TF) of a Gauss neuron under various violations of the equivalence condition of its receiving loops were investigated. It was shown that the imbalance of the self or Josephson inductances of the neuron’s receiving arms leads to a “tilt” in the TF. The distortion of the TF shape in these cases is somewhat different, which provides an opportunity for visual diagnostics of experimental sample faults. The imbalance of the input signal results in the tilting of the baseline, which is not observed in other cases. Comparison with the experiment indicates the presence of input imbalance, which can arise even in a symmetric sample design due to the direct interaction between the input and readout elements.

## Data Availability

All data that supports the findings of this study is available in the published article and/or the supporting information of this article.
